# Distinguishing types and severity of pediatric pneumonia using modified lung ultrasound score

**DOI:** 10.3389/fped.2024.1411365

**Published:** 2024-08-05

**Authors:** Wen Xie, Junxian Ruan, Qiuxia Jiang, Jingyang Zheng, Weiru Lin, Guorong Lyu

**Affiliations:** ^1^ Department of Ultrasound, Quanzhou Maternity and Children’s Hospital, Quanzhou, China; ^2^ Department of Pneumology, Quanzhou Maternity and Children’s Hospital, Quanzhou, China; ^3^ The Headmaster's Office, Quanzhou Medical College, Quanzhou, China; ^4^ Department of Ultrasound, Second Afﬁliated Hospital of Fujian Medical University, Quanzhou, China

**Keywords:** modified lung ultrasound score, pneumonia, ultrasound, children, microbiology

## Abstract

**Objective:**

This study aimed to investigate the diagnostic utility of the modified lung ultrasound score (MLUS) in distinguishing between Mycoplasma pneumonia and viral pneumonia in children and evaluate their severity.

**Methods:**

A prospective collection of 137 suspected cases of community-acquired pneumonia in children admitted to the Quanzhou Maternity and Children's Hospital in Quanzhou City, Fujian Province, from January 2023 to December 2023 constituted the study cohort. All patients underwent lung ultrasound examinations, and MLUS scores were assigned based on ultrasound findings, including pleural lines, A-lines, B-lines, and lung consolidations. Based on the pathogenic results, the patients were categorized into the Mycoplasma pneumonia (74 cases) and viral pneumonia (63 cases) groups. The severity was classified as mild (110 cases) or severe (27 cases). The diagnostic value of MLUS for Mycoplasma pneumonia and viral pneumonia in children was analyzed.

**Results:**

(1) MLUS scores were significantly different between the Mycoplasma pneumonia (15, 10–21) and viral pneumonia (8, 5–16) groups (*P* = 0.002). ROC curve analysis indicated that using a cut-off value of 11, MLUS exhibited a sensitivity of 70.3%, specificity of 58.7%, and an area under curve (AUC) of 0.653 for diagnosing Mycoplasma pneumonia. Furthermore, large-area lung consolidation on ultrasound images demonstrated good diagnostic performance for predicting Mycoplasma pneumonia, with an AUC of 0.763, a sensitivity of 71.6%, and a specificity of 81.0%. (2) MLUS scores were significantly different between the mild pneumonia (10.5, 5–17) and severe pneumonia (21, 16–29) groups (*P* < 0.001). ROC curve analysis using a cut-off value of 16 showed a sensitivity of 77.8%, specificity of 73.6%, and AUC of 0.818 for diagnosing severe pneumonia. Multivariate regression analysis revealed that both MLUS and white blood cell count were independent factors influencing the severity. The constructed nomogram model demonstrated robust stability with a sensitivity of 85.2%, a specificity of 74.5%, and an AUC of 0.858 for predicting severe childhood pneumonia.

**Conclusion:**

MLUS, coupled with ultrasound signs of large-area lung consolidation, had reference significance for the differential diagnosis of Mycoplasma pneumonia and viral pneumonia in children and can be a preliminary assessment of the severity of viral pneumonia or mycoplasma pneumonia in children.

## 1 Introduction

Pneumonia is a prevalent infectious respiratory disease in children, with a substantial proportion of cases falling under the category of community-acquired pneumonia (CAP). CAP accounts for 14% of the total deaths in children below the age of five, contributing to approximately 800,000 child fatalities each year (1). Pathogens associated with CAP encompass viruses, bacteria, and Mycoplasma, with Mycoplasma being the most frequently detected pathogen in hospitalized children aged ≥5 with CAP (2). The proportion of Mycoplasma pneumonia and refractory Mycoplasma pneumonia infections among severe pneumonia cases have been escalating in recent years. Despite systematic drug interventions, certain children experience health deterioration and prolonged illness owing to various factors. The current definitive diagnosis of Mycoplasma pneumonia relies primarily on mycoplasma culture, serological examination, and molecular biology. However, mycoplasma culture is time-consuming, serological examinations exhibit low sensitivity, and molecular biology diagnosis requires sophisticated equipment and technical expertise (3). Respiratory viruses are the predominant pathogens causing CAP in children under 5 years (4). Among these, respiratory syncytial virus (RSV) results in 2% mortality in children under 5 years and 3.6% mortality in infants aged 28 days to 6 months (5). Diagnosing respiratory viral infections depends primarily on nucleic acid detection; however, this approach yields a high false-negative rate. Although patients with Mycoplasma pneumonia and viral pneumonia present similar clinical symptoms, their treatment and prognosis differ significantly. Hence, early differentiation and standardized treatment of Mycoplasma pneumonia and viral pneumonia are crucial to improve outcomes.

In recent years, the field of lung ultrasonography has witnessed rapid development and widespread application in the evaluation of pulmonary diseases in neonates and children. Despite the overlapping clinical symptoms, laboratory tests, and imaging findings between Mycoplasma pneumonia and viral pneumonia, distinct characteristics are still evident in lung ultrasound manifestations (6). The features of viral pneumonia on ultrasound predominantly include abnormal pleural lines, B-lines, diffused B-lines, and small-area lung consolidations. Contrastingly, Mycoplasma pneumonia is more likely to exhibit large-area lung consolidations, bronchial signs, and pleural effusion. Research suggests that lung ultrasound can serve as a sensitive detection tool and aid in the etiological diagnosis of childhood CAP (7). Although multiple lung consolidations may be detected in patients with viral or Mycoplasma pneumonia, the depth of lung consolidations in Mycoplasma pneumonia (median: 20 mm) surpasses that in viral pneumonia (median: 15 mm) (8). Lung ultrasound distinguishes the ultrasound features of pneumonia caused by different pathogens and is associated with pneumonia severity. Evaluating the size of ultrasound-consolidated lung lesions aids in assessing the severity of pneumonia in children, thereby offering a basis for clinical treatment plans (9).

The quantitative assessment of pulmonary diseases using lung ultrasound represents a recent advancement. Although the literature on lung ultrasound is limited, lung ultrasound (LUS) scores quantify the severity and outcomes of pulmonary diseases and differentiate between viral and bacterial pneumonia. In patients with COVID-19 pneumonia, LUS significantly increases with disease severity (10). Tung-Chen et al. (11) employed a 12-zone lung ultrasound score method to analyze 30 cases of severe acute respiratory syndrome pneumonia caused by type 2 coronavirus and 10 cases of bacterial pneumonia. A cut-off value of 10 was used to distinguish between bacterial and viral pneumonia. Our team previously employed the modified lung ultrasound score (MLUS) to quantitatively assess changes in the condition and treatment effects of neonatal respiratory distress and achieved positive outcomes (12, 13). Currently, reports on MLUS usage to differentiate between Mycoplasma pneumonia and viral pneumonia are scarce. However, some researchers have used chest CT to distinguish between COVID-19 and Mycoplasma pneumonia. Imaging manifestations reveal that the COVID-19 pneumonia lesions are generally located in the peripheral posterior region, whereas Mycoplasma pneumonia lesions are distributed along the bronchi (14). We speculate that MLUS may be employed to quantitatively score and differentiate between Mycoplasma pneumonia and viral pneumonia and assess their severity based on this.

Therefore, this study aimed to use MLUS to differentiate between Mycoplasma pneumonia and viral pneumonia early and to quantitatively assess the severity of childhood pneumonia to promote the MLUS application.

## 2 Materials and methods

### 2.1 Study subjects

This prospective study was conducted from January 2023 to December 2023 at Quanzhou Maternity and Children's Hospital in Quanzhou, Fujian, China. Pediatric patients with experimental pneumonia were included based on the following diagnostic criteria for pneumonia:

1. Clinical diagnosis and severity classification of pneumonia referred to the “Management of Community-Acquired Pneumonia in Infants and Children Over 3 Months: Guidelines of the Pediatric Infectious Diseases Society and the Clinical Practice Guidelines of the Infectious Diseases Society of America” (15).
2. Patients with positive Mycoplasma pneumonia detection was included in the Mycoplasma pneumonia group, and those with positive viral detection were included in the viral pneumonia group.
3. All patients underwent lung ultrasound and chest CT examinations.

**Figure 1:**
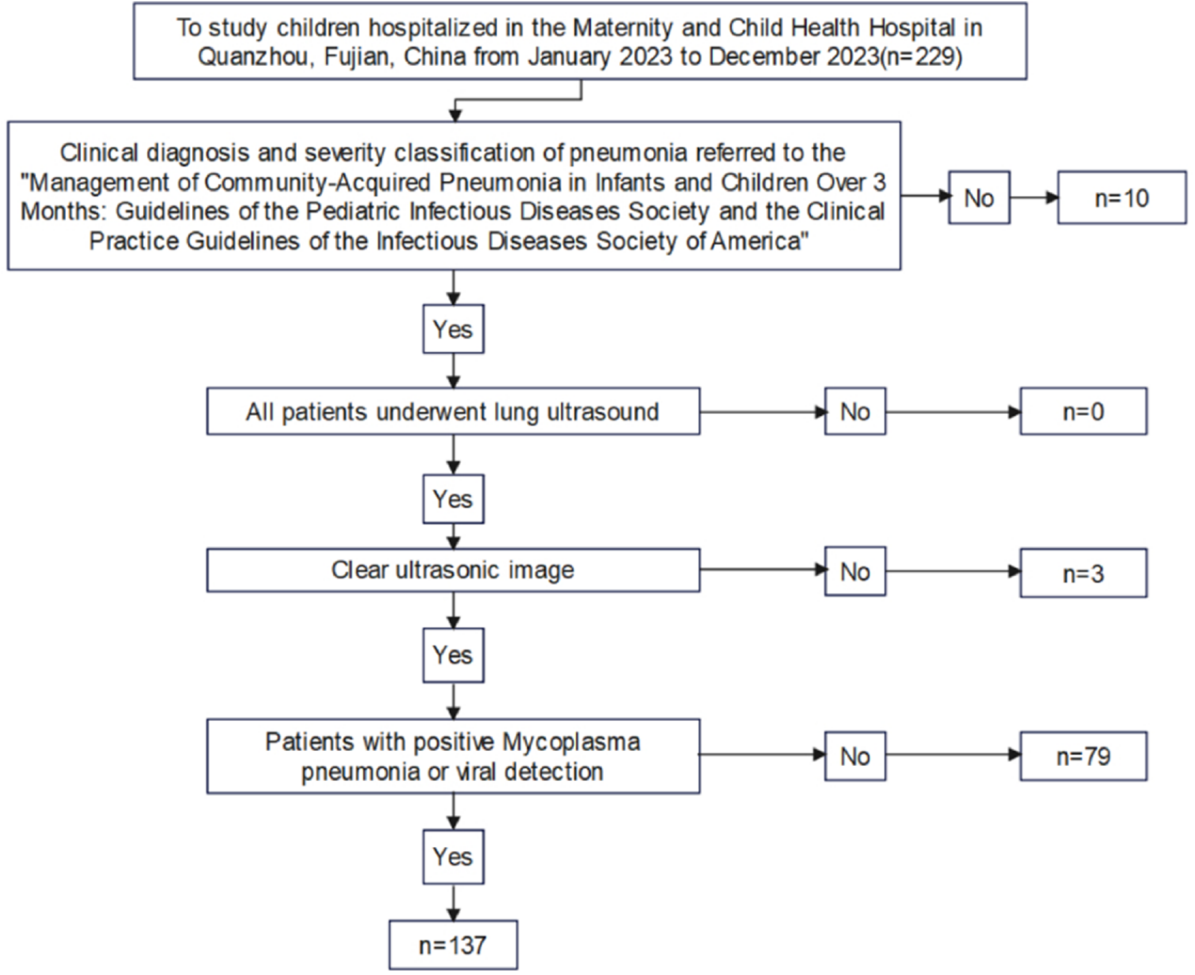
Flow chart of inclusion and exclusion of subjects.

The exclusion criteria included pneumonia induced by foreign body inhalation, severe immunosuppression, complex congenital diseases, and mixed infections with multiple pathogens. Based on the microbiological results of the children as the gold standard, 137 cases comprising 72 males and 65 females, with ages ranging from 0.25 to 12 years (mean age: 4.891 ± 2.926 years) were included. The participants were divided into mycoplasma pneumonia group (74 patients) and viral pneumonia group (63 patients). The diagnosis of mycoplasma pneumonia is made by combined testing, including nucleic acid testing (detection of Mycoplasma pneumoniae DNA or RNA by PCR technique in throat swab, sputum, or bronchoalveolar lavage fluid samples from patients) and serological testing (detection of specific antibodies in blood samples from patients).Viral pneumonia is diagnosed by nucleic acid testing. Based on the severity of the disease, the children were further classified into mild (110 cases) and severe (27 cases) groups. Severe pneumonia is defined as severe ventilation dysfunction or Intrapulmonary and extrapulmonary complications in children with pneumonia. This includes one or more of the following: poor general health; disturbance of consciousness; purple skin; rapid breathing; hypoxemia; high fever; dehydration; severe chest x-ray or chest CT findings; as well as extrapulmonary complications. This study was approved by the Quanzhou Maternity and Children's Hospital Ethics Committee (Ethics Review No. 112 of 2023). Informed consent was obtained from each child's guardian. Figure 1 illustrates the inclusion and exclusion processes of the study participants.

### 2.2 Instruments and methods

A GE Voluson E8 color Doppler ultrasound machine was used with an 11 MHz linear array probe and a 5 MHz convex array probe. The patients were positioned in the supine, lateral, prone, and sitting positions. The linear array probe was placed vertically along the intercostal space, longitudinally and transversely, from top to bottom and right to left. The convex array probe was used below the ribs to scan along the rib edge, and each area was evaluated individually. The ultrasound score for each region was recorded, with the most severe ultrasound score and the presence of B-lines, large-area lung consolidations, small-area lung consolidations, and pleural effusion. Each patient was examined by two radiologists with >five years of experience in lung ultrasound examination. The collected lung ultrasound images were then stored and scored by another senior radiologist to obtain a score.

**Figure 2:**
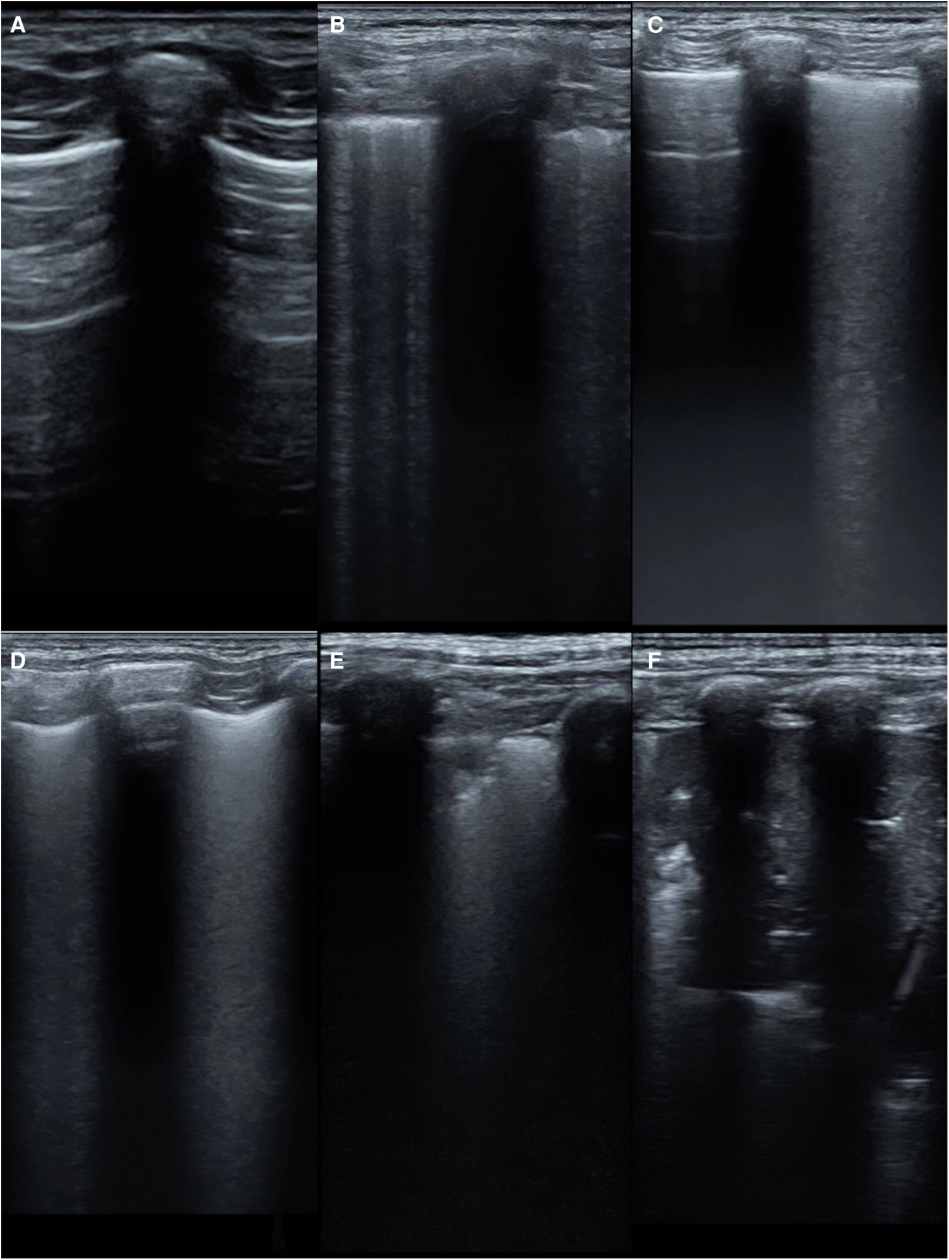
The scoring criteria for the modified lung ultrasound images. (**A**) A-line is dominant, <3 non-fused B-lines(0 point); (**B**) multiple non-fused B-lines(1 point); (**C**) dense, partially fused B-lines(2 points); (**D**) completely fused B-lines(3 points) (**E**) abnormal pleural line with a small range (depth <1 cm) of subpleural lung consolidation(4 points); (**F**) abnormal pleural line with large-area (depth >1 cm) lung consolidation(5 points).

The modified lung ultrasound score (MLUS) was determined using a method developed by our team (12, 13), utilizing a 14-zone approach. The lungs were divided into seven regions on each side, using lines drawn from the sternal edge, anterior axillary line, posterior axillary line, posterior midline, and connecting nipples, resulting in a total score of 70. The scoring criteria for the modified lung ultrasound images are shown in Figure 2. A score of 0 indicated that the A-lines were predominant, with scattered (<3) B-lines. A score of 1 indicated that multiple non-fused B-lines. A score of 2 indicated that dense, partially fused B-lines.A score of 3 indicated that completely fused B-lines. A score of 4 indicated abnormal pleural lines with small-area (<1 cm depth) subpleural lung consolidations. A score of 5 indicated abnormal pleural lines with large-area (>1 cm depth) lung consolidations.

### 2.3 Statistical analysis

Statistical software (SPSS 27.0) was used for all analyses. For normally distributed quantitative data, the results are presented as the mean, and for non-normally distributed data, the results are presented as the median (IQR). The Mann–Whitney *U*-test was used to compare the two groups. Count data were presented as *n* (%), and the chi-square test, or Fisher's exact test, was used to compare groups. Logistic regression was used to analyze factors influencing disease severity. The ROC curve was used to evaluate the predictive value of the scores and ultrasound signs, and the DeLong test was used to compare the predictive values. Construct a column chart using the “rms” package in R software 4.3.2, and evaluate the model fit through calibration curves. Statistical significance was set at *P* < 0.05.

## 3 Results

### 3.1 Comparison of the Mycoplasma pneumonia and viral pneumonia groups

#### 3.1.1 Comparison of clinical and ultrasound indicators between the Mycoplasma pneumonia and viral pneumonia groups

Differences in age, fever, wheezing, white blood cell count, modified lung ultrasound score, large-area consolidations, pleural effusion, and lesion location were statistically significant in Mycoplasma pneumonia and viral pneumonia groups (*P* < 0.05, Table 1). The mean MLUS score in the Mycoplasma pneumonia group was 15 (10,21), which was higher than that in the viral pneumonia group (8, 5, 16). The Mycoplasma pneumonia group was more likely to exhibit large-area lung consolidations on ultrasound than the viral pneumonia group (*P* < 0.05).Chest CT examination was positive for pneumonia in all patients.

**Table 1:** Comparison of indicators between the mycoplasma pneumonia and viral pneumonia groups.

	Mycoplasma pneumonia group (*n* = 74)	viral pneumonia group (*n* = 63)	*X^2^/z*	*P*
Age (years)	6 (4.75,8)	3.25 (1,4.75)	−6.269	0.000
Gender, *n* (%)			0.014	0.904
Female	36 (48.6)	30 (47.6)		
Male	38 (51.4)	33 (52.4)		
Fever, *n* (%)			13.813	0.000
No	9 (12.2)	25 (39.7)		
Yes	65 (87.8)	38 (60.3)		
Cough and sputum, *n* (%)			–	0.210
No	0 (0)	2 (3.2)		
Yes	74 (100)	61 (96.8)		
Wheeze, *n* (%)			32.500	0.000
No	70 (94.6)	33 (52.4)		
Yes	4 (5.4)	30 (47.6)		
Increased c-reactive protein, *n* (%)			0.756	0.384
No	30 (40.5)	21 (33.3)		
Yes	44 (59.5)	42 (66.7)		
White blood cell counts (×10^9^/L)	8.5 (5.9,10.8)	10 (7.5,14.8)	−2.613	0.009
MLUS	15 (10,21)	8 (5,16)	−3.085	0.002
Large-area lung consolidation, *n* (%)			37.722	0.000
No	21 (28.4)	51 (81.0)		
Yes	53 (71.6)	12 (19.0)		
Small-area lung consolidation, *n* (%)			1.727	0.189
No	37 (50.7)	39 (61.9)		
Yes	36 (49.3)	24 (38.1)		
Pleural effusion, *n* (%)			5.951	0.015
No	55 (74.3)	57 (90.5)		
Yes	19 (25.7)	6 (9.5)		
B-lines, *n* (%)			–	–
No	0 (0)	0 (0)		
Yes	74 (100)	63 (100)		
Lesion location, *n* (%)			22.385	0.000
Left lung	7 (9.5)	1 (1.6)		
Right lung	26 (35.1)	5 (7.9)		
Bilateral pulmonary	41 (55.4)	57 (90.5)		

#### 3.1.2 Prediction of Mycoplasma pneumonia using modified lung ultrasound score, large-area lung consolidations, and pleural effusion

**Figure 3:**
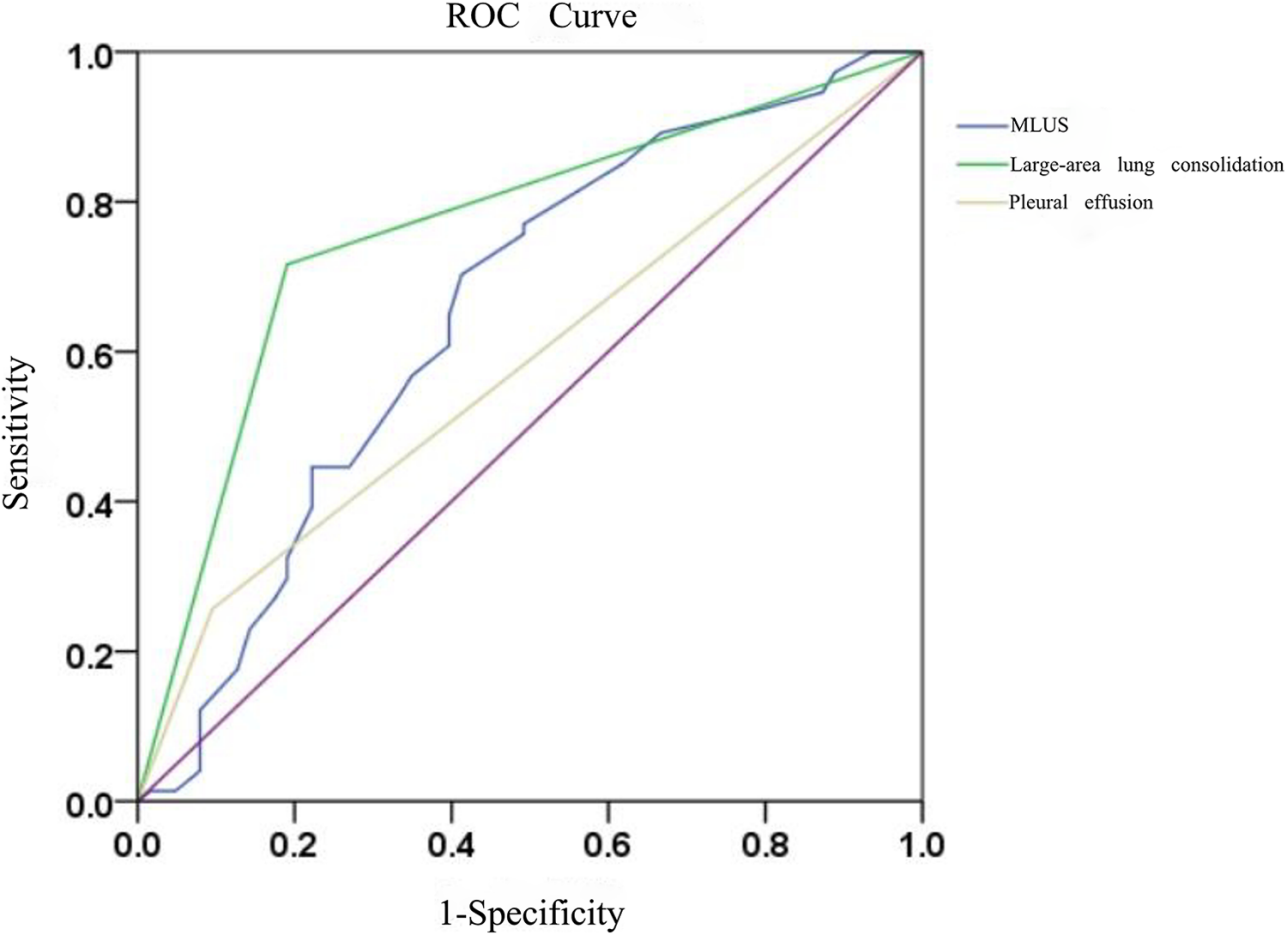
The diagnostic efficacy of modified lung ultrasound score and large-area lung consolidation in predicting mycoplasma pneumonia.

The results of the ROC curve analysis (Figure 3) showed that the modified lung ultrasound score and large-area lung consolidation had predictive values for Mycoplasma pneumonia. When the modified lung ultrasound score was ≥11 points, the prediction of Mycoplasma pneumonia had an AUC of 0.653, with a sensitivity of 70.3% and specificity of 58.7%. The presence of large-area lung consolidations had good diagnostic efficiency for predicting Mycoplasma pneumonia, with an AUC of 0.763, a sensitivity of 71.6%, and a specificity of 81.0%. The DeLong test indicated that the difference in predictive value between the modified lung ultrasound score and large-area lung consolidations for Mycoplasma pneumonia was statistically significant (Z = 2.943, *P* < 0.05). Similarly, the comparison between large-area lung consolidation and pleural effusion was statistically significant (Z = 4.376, *P* < 0.05), whereas no significant difference was observed between the modified lung ultrasound score and pleural effusion (Z = 1.493, *P* > 0.05; Table 2).

**Table 2:** Prediction of mycoplasma pneumonia by modified lung ultrasound score, large-area lung consolidation and pleural effusion.

	AUC	95% CI	*P*	Sensitivity (%)	Specificity (%)	Cut-off value
MLUS	0.653^a^	0.559–0.747	0.002	70.3	58.7	11
Large-area lung consolidation	0.763	0.610–0.845	0.000	71.6	81.0	–
Pleural effusion	0.581^a^	0.486–0.676	0.104	74.3	90.5	–

MLUS, modified lung ultrasound score.

^a^ *P*: Compared with large-area lung consolidation, *P*<0.05.

### 3.2 Comparison of clinical and ultrasound indicators between mild pneumonia and severe pneumonia groups

#### 3.2.1 Comparison of clinical and ultrasound indicators between the mild and severe pneumonia groups and construction of a predictive model

The comparison between the mild and severe groups showed statistically significant differences in age, fever, wheezing, CRP, white blood cell count, MLUS score, pleural effusion, and pathogens (*P* < 0.05; Table 3), suggesting the potential factors influencing severity. The MLUS score in the severe group was 21 (16,29), which was higher than the 10.5 (5,17) in the mild group (Z = −5.084, *P* < 0.05).

**Table 3:** Comparison of clinical and ultrasound indicators between mild pneumonia and severe pneumonia groups.

	Mild pneumonia pneumonia group (*n* = 110)	Severe pneumonia group (*n* = 27)	*X^2^/z*	*P*
Age (years)	5.29 (3.33,7)	3 (0.83,5.08)	−3.376	0.001
Gender, *n* (%)			0.000	0.997
Female	53 (48.2)	13 (48.1)		
Male	57 (51.8)	14 (51.9)		
Fever, *n* (%)			4.570	0.033
No	23 (20.9)	11 (40.7)		
Yes	87 (79.1)	16 (59.3)		
Cough and sputum, *n* (%)			–	0.356
No	1 (0.9)	1 (3.7)		
Yes	109 (99.1)	26 (96.3)		
Wheeze, *n* (%)			13.172	0.000
No	90 (81.8)	13 (48.1)		
Yes	20 (18.2)	14 (51.9)		
Increased c-reactive protein, *n* (%)			5.036	0.025
No	46 (41.8)	5 (18.5)		
Yes	64 (58.2)	22 (81.5)		
White blood cell counts (×10^9^/L)	8.5 (6.2,11.4)	11.6 (9.2,20.5)	−3.658	0.000
MLUS	10.5 (5,17)	21 (16,29)	−5.084	0.000
Large-area lung consolidation, *n* (%)			1.882	0.170
No	61 (55.5)	11 (40.7)		
Yes	49 (44.5)	16 (59.3)		
Small-area lung consolidation, *n* (%)			0.001	0.970
No	61 (56.0)	15 (55.6)		
Yes	48 (44.0)	12 (44.4)		
Pleural effusion, *n* (%)			3.947	0.047
No	94 (85.5)	18 (66.7)		
Yes	16 (14.5)	9 (33.3)		
B-lines, *n* (%)			–	–
No	0 (0)	0 (0)		
Yes	110 (100)	27 (100)		
Lesion location, *n* (%)			3.747	0.154
Left lung	8 (7.3)	0 (0)		
Right lung	27 (24.5)	4 (14.8)		
Bilateral pulmonary	75 (68.2)	23 (85.2)		
Pathogen, *n* (%)			8.05	0.005
Mycoplasma pneumoniae	66 (60.0)	8 (29.6)		
Virus	44 (40.0)	19 (70.4)		

#### 3.2.2 Construction of a line chart for predicting the risk of severe pneumonia using modified lung ultrasound score and white blood cell count

**Figure 4:**
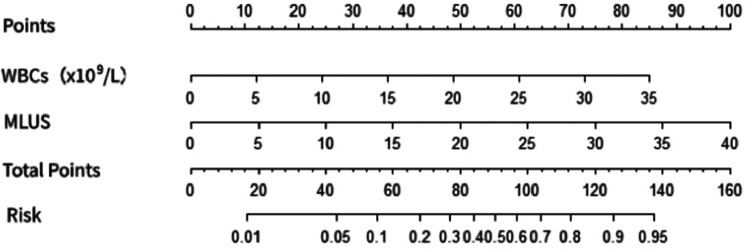
Nomogram of the prediction of severe pneumonia by modified lung ultrasound score and white blood cell count.

**Figure 5:**
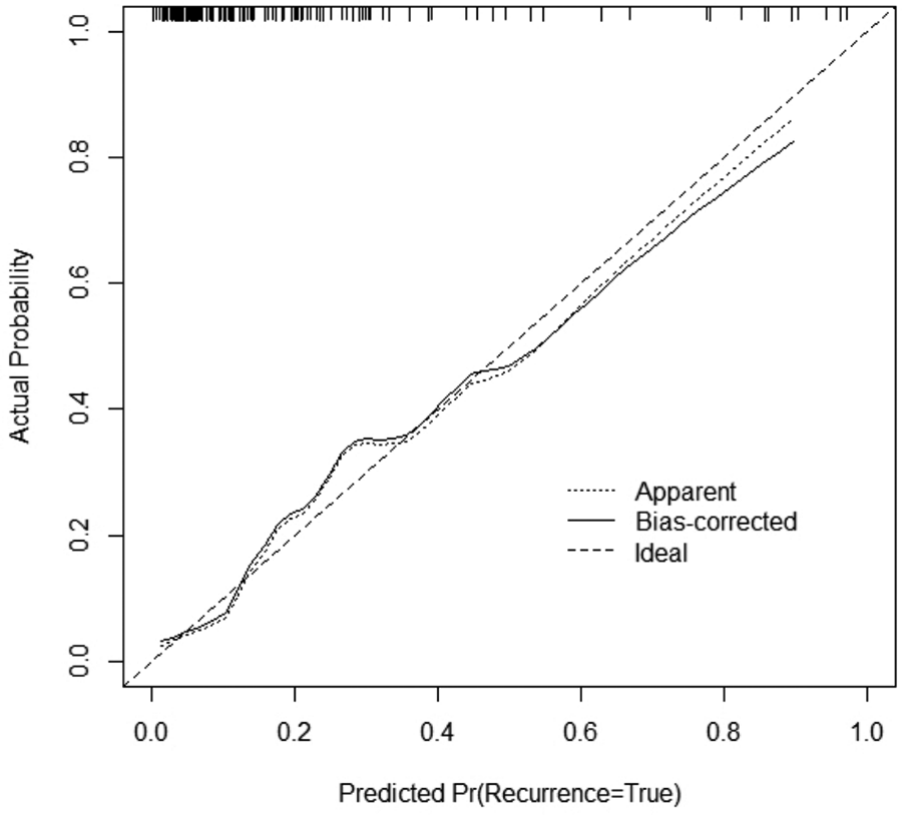
Calibration curve of the nomogram.

**Figure 6:**
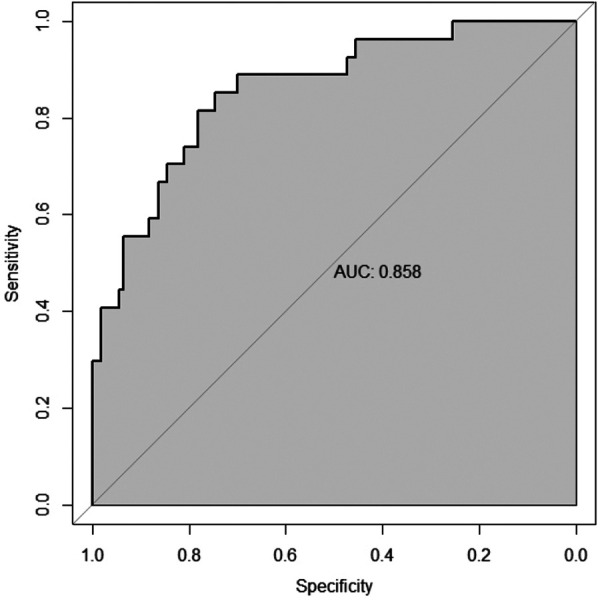
Differentiation degree of the nomogram.

The multivariate logistic regression analysis (Table 4) results indicated that MLUS and white blood cell count were independent risk factors for severity. Both higher MLUS scores and higher white blood cell counts were associated with a higher risk of severity. The other indicators were not independent risk factors for severe pneumonia. A nomogram constructed using these two factors is shown in Figure 4. The C-index of the nomogram model was 0.858, indicating good clinical effectiveness. Internal validation using the bootstrap method showed good consistency between the actual observations and line chart predictions, with an absolute error of 0.022, indicating high model calibration (Figure 5). The ROC curve evaluation of the model's predictive value for severity showed an AUC (95% CI) of 0.858 (0.779–0.936), indicating a certain predictive value for prognosis. When the optimal cut-off value was selected, the sensitivity and specificity were 85.2% and 74.5%, respectively, suggesting good predictive model performance (Figure 6).

**Table 4:** Results of logistic regression analysis of influencing factors of severe pneumonia.

	*B*	*S.E.*	*Wald*	*P*	*OR*	95% CI	
						Inferior limit	Superior limit
Age	−0.074	0.146	0.256	0.613	0.929	0.698	1.236
Fever	−0.924	0.970	0.907	0.341	0.397	0.059	2.658
Wheeze	1.168	1.054	1.228	0.268	3.215	0.408	25.365
Increased c-reactive protein	0.513	0.792	0.420	0.517	1.670	0.354	7.882
White blood cell counts	0.113	0.055	4.212	0.040	1.119	1.005	1.247
MLUS scores	0.208	0.053	15.231	0.000	1.231	1.109	1.367
Pleural effusion	1.130	0.827	1.867	0.172	3.095	0.612	15.655
Pathogen	1.582	1.014	2.433	0.119	4.866	0.666	35.539

#### 3.2.3 Prediction of pneumonia severity using modified lung ultrasound score

**Figure 7:**
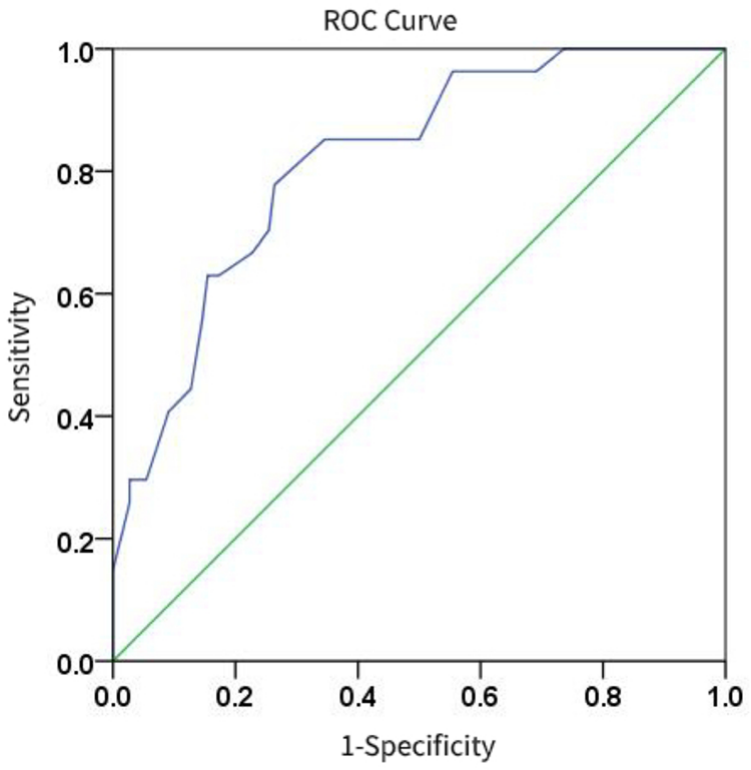
The diagnostic efficacy of modified lung ultrasound score in predicting severe pneumonia.

The results of the ROC curve analysis (Figure 7) showed that the modified lung ultrasound score had a certain predictive value for severity. When the score was ≥16 points, the likelihood of severe pneumonia increased, showing good diagnostic efficiency with an AUC of 0.818, sensitivity of 77.8%, and specificity of 73.6%.

## 4 Discussion

Our study found that MLUS scoring effectively distinguished between mild and severe pneumonia, consistent with the results of previous studies (16). The severity of the condition increased along with an increase in the lung ultrasound score, indicating poorer lung ventilation. During pneumonia, a reduced lung ventilation function, inflammatory exudation in the lung interstitium and alveoli, and increased water content in the lung lead to increased high-echo B-lines from the pleura to the deep lungs on ultrasound, indicating fusion. With increasing water in the lung, lung ventilation disappeared, and ultrasound showed lung consolidation. Different images may be observed on ultrasound as the lung tissue undergoes varying degrees of deaeration. Therefore, evaluation of the severity of pneumonia using ultrasound is feasible. Using a 14-zone approach increases the coverage of both lungs and provides a more detailed assessment by exploring bilateral lung bases. Pneumonia can cause increased lung water, gravitational settling of lung water, and increased probing of the lung base, resulting in a more detailed score. Thus, MLUS can be used for the early quantitative assessment of pneumonia severity. Early intervention should be emphasized when the MLUS score is ≥16 points. The results of this study further suggest that the model for distinguishing between mild and severe childhood pneumonia constructed using MLUS scores and white blood cell counts is robust and has good diagnostic efficiency. Therefore, it can be used for early prediction and intervention to reduce adverse outcomes of childhood pneumonia.

This is the first study to use MLUS for the differential diagnosis of childhood Mycoplasma pneumonia and viral pneumonia. The results indicate that MLUS can differentiate between Mycoplasma pneumonia and viral pneumonia, with a sensitivity of 70.3% and specificity of 58.7% when the MLUS score is ≥11 points. Although the diagnostic efficiency of MLUS for distinguishing between Mycoplasma pneumonia and viral pneumonia is slightly lower than that of ultrasound signs of lung consolidation (depth greater than 10 mm), research suggests that in the early stages of viral pneumonia or Mycoplasma pneumonia, large or multilobar consolidation is less commonly seen on tracheal imaging (17). Therefore, MLUS combined with large-area lung consolidation remains necessary to distinguish between Mycoplasma pneumonia and viral pneumonia. Adhesive proteins on Mycoplasma pneumonia allow the bacterium to adhere to the respiratory mucosal epithelial cilia for self-replication, disrupt respiratory defense functions, and directly damage the respiratory epithelium, causing bronchial mucosal edema, thickening of the bronchial wall, and other signs of bronchitis (18). Therefore, the lesions followed the course of the bronchus, and ultrasound signs showed large-area lung consolidation. In contrast, viral pneumonia mainly presents with diffuse interstitial inflammation and alveolar damage, including apoptosis and shedding of alveolar epithelial cells, formation of transparent pulmonary membranes, significantly widened interlobular spaces, and infiltration of inflammatory cells, including lymphocytes and monocytes (19). Therefore,ultrasound signs of viral pneumonia commonly include increased B-lines and small-area lung consolidation.

In clinical practice, lung ultrasound can be used to distinguish severe pneumonia from mild pneumonia. But for the etiological diagnosis of pneumonia infection, there is a need for microbiological examination. The results of this study indicate that MLUS can be used to distinguish mycoplasma pneumonia from viral pneumonia. We also found that the ultrasound signs of extensive pneumonia consolidation were effective in diagnosing mycoplasma pneumonia, with AUC = 0.763, sensitivity 71.6%, and specificity 81.0%. Mycoplasma pneumonia is more likely to be diagnosed when children are older than 5 years old. It usually occurs in autumn and winter. The onset is slow, the first dry cough is followed by expectoration, there is no rales on lung ausculation, the lung ultrasound shows large-scale consolidation, and the modified lung ultrasound score is more than 11. When children with suspected pneumonia are younger than 5 years old, onset in winter and spring, sudden onset, wheezing, wet rales on lung auscultation, small range of lung consolidation on lung ultrasound, and the modified lung ultrasound score is less than 11, we are more inclined to diagnose viral pneumonia. However, the current study mainly included cases of mycoplasma pneumonia and viral pneumonia, and bacterial pneumonia was not included in the scope of the study. Previous studies have shown that the depth of consolidation of bacterial pneumonia is greater than that of mycoplasma pneumonia and viral pneumonia. For example, Streptococcus pneumoniae often causes lobar pneumonia with a large range of lesions. In the future, we will further investigate the differences in lung ultrasound caused by more types of pathogenic pathogens.

This study had some limitations. The 14-zone method used in the lung area only divides large and small consolidations based on depth without measuring the total number of lung consolidations in each zone. In addition, deep lesions in the lung may not be visible on ultrasound due to gas obstruction, and the condition of the superficial lung may not reflect the overall condition of the lung disease. The acquisition and interpretation of lung ultrasound images depend on the operator's skill and proficiency, introducing some subjectivity to the scoring results.

## 5 Conclusion

MLUS can effectively quantify the severity of childhood Mycoplasma pneumonia and viral pneumonia, and there are some references which, when combined with ultrasound signs of large-area lung consolidations,can help distinguish between these conditions. Its simple evaluation method, lack of radiation damage, and role as a vital auxiliary examination tool make it advantageous for clinical diagnosis and treatment.

## Data Availability

The raw data supporting the conclusions of this article will be made available by the authors, without undue reservation.

## References

[1] GBD 2017 Lower Respiratory Infections Collaborators. Quantifying risks and interventions that have affected the burden of lower respiratory infections among children younger than 5 years: an analysis for the global burden of disease study 2017. Lancet Infect Dis. (2020) 20(1):60–79. 10.1016/S1473-3099(19)30410-4 31678026 PMC7185492

[2] Kutty PK Jain S Taylor TH Bramley AM Diaz MH Ampofo K Mycoplasma pneumoniae among children hospitalized with community-acquired pneumonia. Clin Infect Dis. (2019) 68(1):5–12. 10.1093/cid/ciy419 29788037 PMC6552676

[3] Kumar S Kumar S. Mycoplasma pneumoniae: among the smallest bacterial pathogens with great clinical significance in children. Indian J Med Microbiol. (2023) 46:100480. 10.1016/j.ijmmb.2023.100480 37741157

[4] Rueda ZV Aguilar Y Maya MA López L Restrepo A Garcés C Etiology and the challenge of diagnostic testing of community-acquired pneumonia in children and adolescents. BMC Pediatr. (2022) 22(1):169. 10.1186/s12887-022-03235-z 35361166 PMC8968093

[5] Li Y Wang X Blau DM Caballero MT Feikin DR Gill CJ Global, regional, and national disease burden estimates of acute lower respiratory infections due to respiratory syncytial virus in children younger than 5 years in 2019: a systematic analysis. Lancet. (2022) 399(10340):2047–64. 10.1016/S0140-6736(22)00478-0 35598608 PMC7613574

[6] Tan G Lian X Zhu Z Wang Z Huang F Zhang Y Use of lung ultrasound to differentiate coronavirus disease 2019 (COVID-19) pneumonia from community-acquired pneumonia. Ultrasound Med Biol. (2020) 46(10):2651–8. 10.1016/j.ultrasmedbio.2020.05.006 32622684 PMC7274602

[7] Harel-Sterling M Diallo M Santhirakumaran S Maxim T Tessaro M. Emergency department resource use in pediatric pneumonia: point-of-care lung ultrasonography versus chest radiography. J Ultrasound Med. (2019) 38(2):407–14. 10.1002/jum.14703 30027608

[8] Berce V Tomazin M Gorenjak M Berce T Lovrenčič B. The usefulness of lung ultrasound for the aetiological diagnosis of community-acquired pneumonia in children. Sci Rep. (2019) 9(1):17957. 10.1038/s41598-019-54499-y 31784642 PMC6884636

[9] Guo Z Zhang X Yuan Y. The value of lung ultrasound in assessing the degree of lesions in children with mycoplasma pneumoniae pneumonia. Am J Transl Res. (2023) 15(3):2175–82. PMID: . 37056819 PMC10086898

[10] Sumbul HE Koc AS Pınar A Aslan MZ Gulumsek E Koca H Modified lung ultrasound score in evaluating the severity of COVID-19 pneumonia. Ultrasound Med Biol. (2021) 47(8):2080–9. 10.1016/j.ultrasmedbio.2021.04.023 34088529 PMC8086809

[11] Tung-Chen Y Giraldo Hernández A Mora Vargas A Dorado Doblado L González Merino PE Valencia Alijo Á Impact of lung ultrasound during the SARS-CoV-2 pandemic: distinction between viral and bacterial pneumonia. Reumatol Clin (Engl Ed). (2022) 18(9):546–50. 10.1016/j.reumae.2021.09.006 35504823 PMC8930392

[12] Jiang QX Shi LJ Shen LY Li XQ Huang RS Chen LJ Application value of a new lung ultrasound scoring method in neonatal respiratory distress syndrome treatment. Ultrasound Med Biol. (2022) 48(2):275–82. 10.1016/j.ultrasmedbio.2021.10.009 34782166

[13] Jiang QX Lyu GR Li XQ Shi LJ Huang RS Shen LY. Evaluation of the efficacy of alveolar surfactant in neonates with respiratory distress syndrome by a new pulmonary ultrasound scoring method. Chin J Med Imaging. (2021) 29(5):464–8. 10.3969/j.issn.1005-5185.2021.05.012

[14] Huo X Xue X Yuan S Zhang D Gao Q Gong T. Early differential diagnosis between COVID-19 and mycoplasma pneumonia with chest CT scan. Zhejiang Da Xue Xue Bao Yi Xue Ban. (2020) 49(4):468–73. 10.3785/j.issn.1008-9292.2020.07.04 32985160 PMC8800730

[15] Bradley JS Byington CL Shah SS Alverson B Carter ER Harrison C The management of community-acquired pneumonia in infants and children older than 3 months of age: clinical practice guidelines by the pediatric infectious diseases society and the Infectious Diseases Society of America. Clin Infect Dis. (2011) 53(7):e25–76. 10.1093/cid/cir531 21880587 PMC7107838

[16] Baciarello M Bonetti A Vetrugno L Saturno F Nouvenne A Bellini V Is lung ultrasound score a useful tool to monitoring and handling moderate and severe COVID-19 patients in the general ward? An observational pilot study. J Clin Monit Comput. (2022) 36(3):785–93. 10.1007/s10877-021-00709-w 33948780 PMC8096129

[17] Pérez Pallarés J Lerenas Bernal F Cabello Jabalquinto MR Jiménez Romero AA. Usefulness of thoracic ultrasound for diagnosis and follow-up of pneumonia. Rev Esp Quimioter. (2022) 35(Suppl 1):21–4. 10.37201/req/s01.04.2022 35488819 PMC9106189

[18] Waites KB Xiao L Liu Y Balish MF Atkinson TP. Mycoplasma pneumoniae from the respiratory tract and beyond. Clin Microbiol Rev. (2017) 30(3):747–809. 10.1128/CMR.00114-16 28539503 PMC5475226

[19] Xu Z Shi L Wang Y Zhang J Huang L Zhang C Pathological findings of COVID-19 associated with acute respiratory distress syndrome. Lancet Respir Med. (2020) 8(4):420–2. 10.1016/S2213-2600(20)30076-X 32085846 PMC7164771

