# Supplementary material for: Distinguishing types and severity of pediatric pneumonia using modified lung ultrasound score
Source: Front Pediatr. 2024 Aug 5;12:1411365. doi: 10.3389/fped.2024.1411365 (PMC11330795; doi:10.3389/fped.2024.1411365)
Supplement: Supplementary file 1 [file Datasheet1.docx]

Supplementary Material

# Supplementary Figures and Tables

## Supplementary Figures


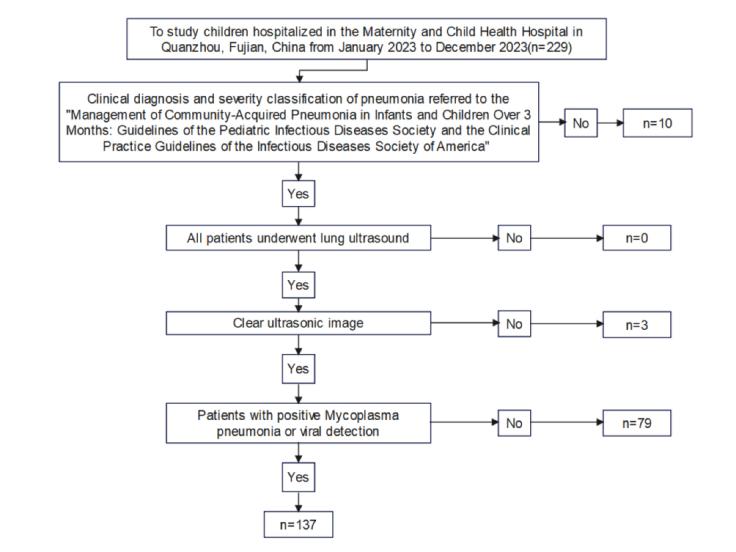


Figure 1. Flow chart of inclusion and exclusion of subjects


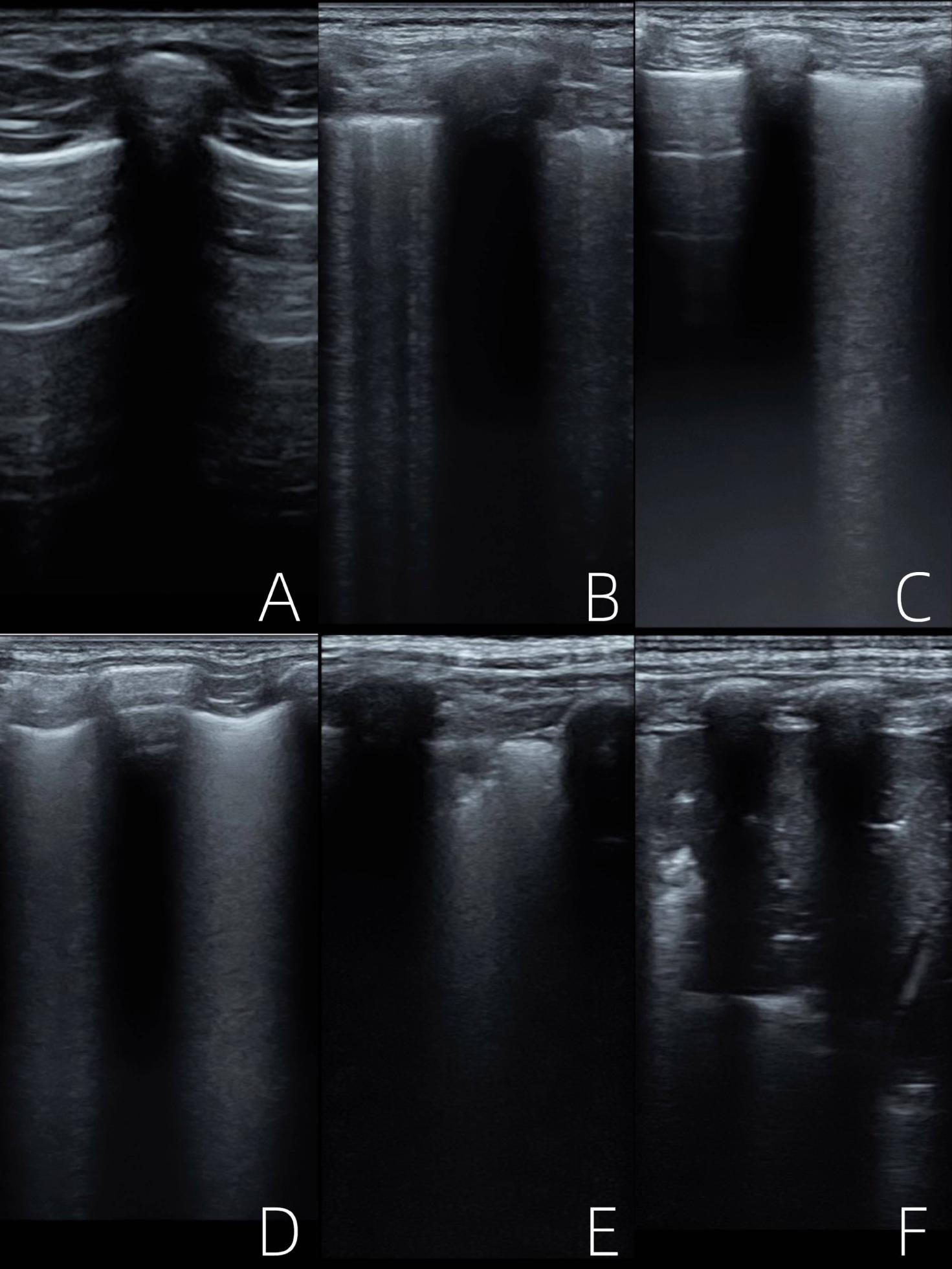


Figure 2.The scoring criteria for the modified lung ultrasound images

A：A-line is dominant, <3 non-fused B-lines(0 point)；B：multiple non-fused B-lines(1 point)；C：dense, partially fused B-lines(2 points)；D：completely fused B-lines(3 points)；E：abnormal pleural line with a small range (depth <1 cm) of subpleural lung consolidation(4 points)；F：abnormal pleural line with large-area (depth >1 cm) lung consolidation(5 points).


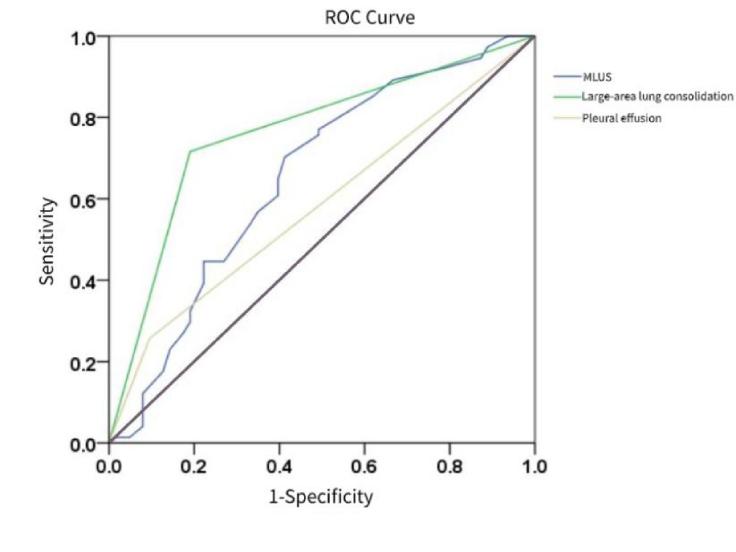


Figure 3. The diagnostic efficacy of modified lung ultrasound score and large-area lung consolidation in predicting mycoplasma pneumonia


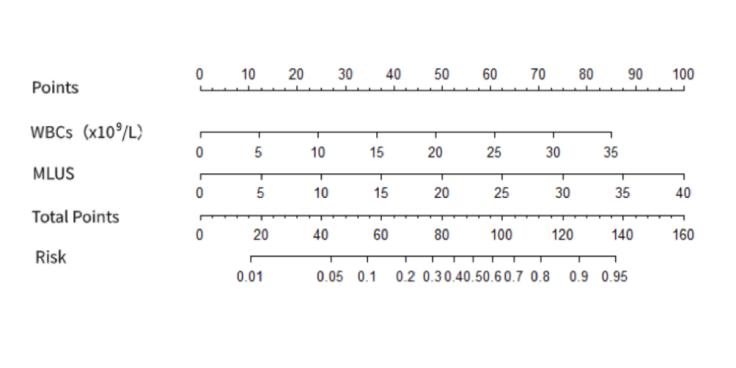


Figure 4. Nomogram of the prediction of severe pneumonia by modified lung ultrasound score and white blood cell count

WBCs=White blood cell counts


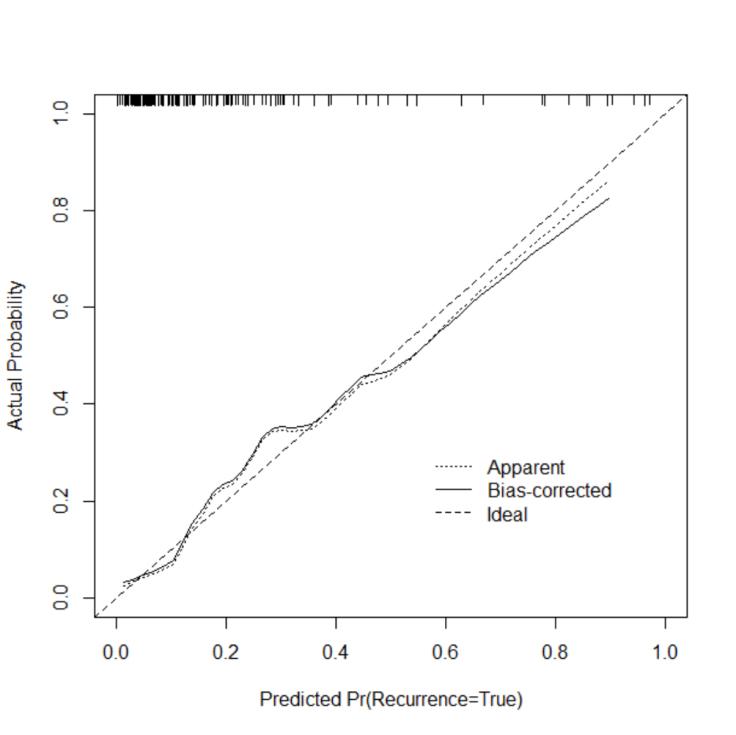


Figure 5. Calibration curve of the nomogram


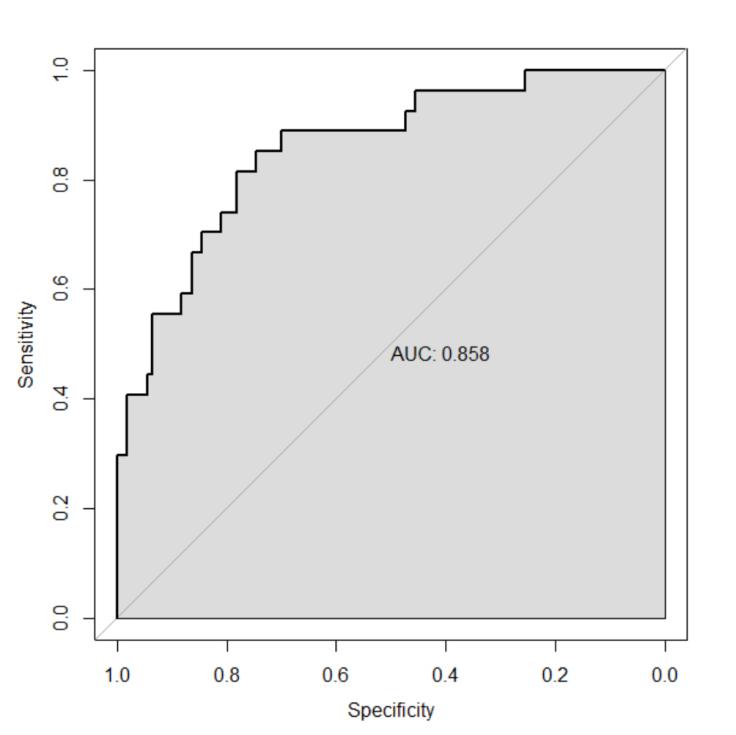


Figure 6. Differentiation degree of the nomogram


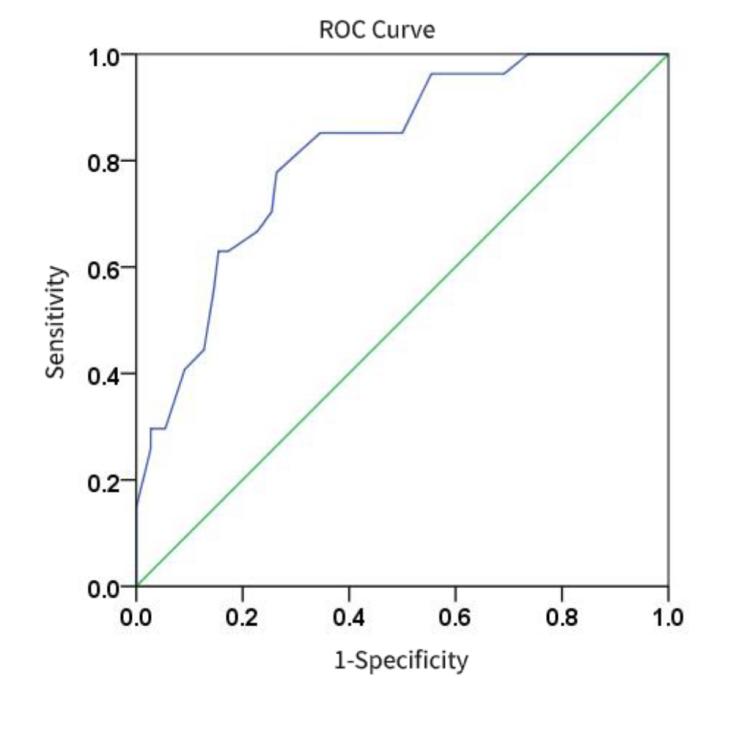


Figure 7. The diagnostic efficacy of modified lung ultrasound score in predicting severe pneumonia

## Supplementary Tables

Table 1: Comparison of indicators between the mycoplasma pneumonia and viral pneumonia groups

|  | Mycoplasma pneumonia group（n=74） | viral pneumonia group（n=63） | *X^2^/z* | *P* |
| --- | --- | --- | --- | --- |
| Age（years） | 6(4.75,8) | 3.25(1,4.75) | -6.269 | 0.000 |
| Gender,n（%） |  |  | 0.014 | 0.904 |
| Female | 36(48.6) | 30(47.6) |  |  |
| Male | 38(51.4) | 33(52.4) |  |  |
| Fever,n（%） |  |  | 13.813 | 0.000 |
| No | 9(12.2) | 25(39.7) |  |  |
| Yes | 65(87.8) | 38(60.3) |  |  |
| Cough and sputum,n（%） |  |  | - | 0.210 |
| No | 0(0) | 2(3.2) |  |  |
| Yes | 74(100) | 61(96.8) |  |  |
| Wheeze,n（%） |  |  | 32.500 | 0.000 |
| No | 70(94.6) | 33(52.4) |  |  |
| Yes | 4(5.4) | 30(47.6) |  |  |
| Increased c-reactive protein,n（%） |  |  | 0.756 | 0.384 |
| No | 30(40.5) | 21(33.3) |  |  |
| Yes | 44(59.5) | 42(66.7) |  |  |
| White blood cell counts（x10^9^/L） | 8.5(5.9,10.8) | 10(7.5,14.8) | -2.613 | 0.009 |
| MLUS | 15(10,21) | 8(5,16) | -3.085 | 0.002 |
| Large-area lung consolidation,n（%） |  |  | 37.722 | 0.000 |
| No | 21(28.4) | 51(81.0) |  |  |
| Yes | 53(71.6) | 12(19.0) |  |  |
| Small-area lung consolidation,n（%） |  |  | 1.727 | 0.189 |
| No | 37(50.7) | 39(61.9) |  |  |
| Yes | 36(49.3) | 24(38.1) |  |  |
| Pleural effusion,n（%） |  |  | 5.951 | 0.015 |
| No | 55(74.3) | 57(90.5) |  |  |
| Yes | 19(25.7) | 6(9.5) |  |  |
| B-lines,n（%） |  |  | - | - |
| No | 0(0) | 0(0) |  |  |
| Yes | 74(100) | 63(100) |  |  |
| Lesion location,n（%） |  |  | 22.385 | 0.000 |
| Left lung | 7(9.5) | 1(1.6) |  |  |
| Right lung | 26(35.1) | 5(7.9) |  |  |
| Bilateral pulmonary | 41(55.4) | 57(90.5) |  |  |

Table 2: Prediction of mycoplasma pneumonia by modified lung ultrasound score, large-area lung consolidation and pleural effusion

|  | AUC | 95%CI | *P* | Sensitivity（%） | Specificity（%） | Cut-off value |
| --- | --- | --- | --- | --- | --- | --- |
| MLUS | 0.653^a^ | 0.559-0.747 | 0.002 | 70.3 | 58.7 | 11 |
| Large-area lung consolidation | 0.763 | 0.610-0.845 | 0.000 | 71.6 | 81.0 | - |
| Pleural effusion | 0.581^a^ | 0.486-0.676 | 0.104 | 74.3 | 90.5 | - |

MLUS=modified lung ultrasound score；^a^*P*：Compared with large-area lung consolidation，*P*＜0.05

Table 3.Comparison of clinical and ultrasound indicators between mild pneumonia and severe pneumonia groups

|  | Mild pneumonia pneumonia group（n=110） | Severe pneumonia group（n=27） | *X^2^/z* | *P* |
| --- | --- | --- | --- | --- |
| Age（years） | 5.29(3.33,7) | 3(0.83,5.08) | -3.376 | 0.001 |
| Gender,n（%） |  |  | 0.000 | 0.997 |
| Female | 53(48.2) | 13(48.1) |  |  |
| Male | 57(51.8) | 14(51.9) |  |  |
| Fever,n（%） |  |  | 4.570 | 0.033 |
| No | 23(20.9) | 11(40.7) |  |  |
| Yes | 87(79.1) | 16(59.3) |  |  |
| Cough and sputum,n（%） |  |  | - | 0.356 |
| No | 1(0.9) | 1(3.7) |  |  |
| Yes | 109(99.1) | 26(96.3) |  |  |
| Wheeze,n（%） |  |  | 13.172 | 0.000 |
| No | 90(81.8) | 13(48.1) |  |  |
| Yes | 20(18.2) | 14(51.9) |  |  |
| Increased c-reactive protein,n（%） |  |  | 5.036 | 0.025 |
| No | 46(41.8) | 5(18.5) |  |  |
| Yes | 64(58.2) | 22(81.5) |  |  |
| White blood cell counts（x10^9^/L） | 8.5(6.2,11.4) | 11.6(9.2,20.5) | -3.658 | 0.000 |
| MLUS | 10.5(5,17) | 21(16,29) | -5.084 | 0.000 |
| Large-area lung consolidation,n（%） |  |  | 1.882 | 0.170 |
| No | 61(55.5) | 11(40.7) |  |  |
| Yes | 49(44.5) | 16(59.3) |  |  |
| Small-area lung consolidation,n（%） |  |  | 0.001 | 0.970 |
| No | 61(56.0) | 15(55.6) |  |  |
| Yes | 48(44.0) | 12(44.4) |  |  |
| Pleural effusion,n（%） |  |  | 3.947 | 0.047 |
| No | 94(85.5) | 18(66.7) |  |  |
| Yes | 16(14.5) | 9(33.3) |  |  |
| B-lines,n（%） |  |  | - | - |
| No | 0(0) | 0(0) |  |  |
| Yes | 110(100) | 27(100) |  |  |
| Lesion location,n（%） |  |  | 3.747 | 0.154 |
| Left lung | 8(7.3) | 0(0) |  |  |
| Right lung | 27(24.5) | 4(14.8) |  |  |
| Bilateral pulmonary | 75(68.2) | 23(85.2) |  |  |
| Pathogen,n（%） |  |  | 8.05 | 0.005 |
| Mycoplasma pneumoniae | 66(60.0) | 8(29.6) |  |  |
| virus | 44(40.0) | 19(70.4) |  |  |

Table 4.Results of Logistic regression analysis of influencing factors of severe pneumonia

|  | *B* | *S.E.* | *Wald* | *P* | *OR* | *95%CI* | |
| --- | --- | --- | --- | --- | --- | --- | --- |
|  |  |  |  |  |  | inferior limit | superior limit |
| Age | -0.074 | 0.146 | 0.256 | 0.613 | 0.929 | 0.698 | 1.236 |
| Fever | -0.924 | 0.970 | 0.907 | 0.341 | 0.397 | 0.059 | 2.658 |
| Wheeze | 1.168 | 1.054 | 1.228 | 0.268 | 3.215 | 0.408 | 25.365 |
| Increased c-reactive protein | 0.513 | 0.792 | 0.420 | 0.517 | 1.670 | 0.354 | 7.882 |
| White blood cell counts | 0.113 | 0.055 | 4.212 | 0.040 | 1.119 | 1.005 | 1.247 |
| MLUS scores | 0.208 | 0.053 | 15.231 | 0.000 | 1.231 | 1.109 | 1.367 |
| Pleural effusion | 1.130 | 0.827 | 1.867 | 0.172 | 3.095 | 0.612 | 15.655 |
| Pathogen | 1.582 | 1.014 | 2.433 | 0.119 | 4.866 | 0.666 | 35.539 |
